# The Production and Perception of Emotionally Expressive Walking Sounds: Similarities between Musical Performance and Everyday Motor Activity

**DOI:** 10.1371/journal.pone.0115587

**Published:** 2014-12-31

**Authors:** Bruno L. Giordano, Hauke Egermann, Roberto Bresin

**Affiliations:** 1 Institute of Neuroscience and Psychology, University of Glasgow, Glasgow, United Kingdom; 2 Audio Communication Group, Technische Universität Berlin, Berlin, Germany; 3 Sound and Music Computing Group, KTH Royal Institute of Technology, Stockholm, Sweden; University of Tuebingen Medical School, Germany

## Abstract

Several studies have investigated the encoding and perception of emotional expressivity in music performance. A relevant question concerns how the ability to communicate emotions in music performance is acquired. In accordance with recent theories on the embodiment of emotion, we suggest here that both the expression and recognition of emotion in music might at least in part rely on knowledge about the sounds of expressive body movements. We test this hypothesis by drawing parallels between musical expression of emotions and expression of emotions in sounds associated with a non-musical motor activity: walking. In a combined production-perception design, two experiments were conducted, and expressive acoustical features were compared across modalities. An initial performance experiment tested for similar feature use in walking sounds and music performance, and revealed that strong similarities exist. Features related to sound intensity, tempo and tempo regularity were identified as been used similarly in both domains. Participants in a subsequent perception experiment were able to recognize both non-emotional and emotional properties of the sound-generating walkers. An analysis of the acoustical correlates of behavioral data revealed that variations in sound intensity, tempo, and tempo regularity were likely used to recognize expressed emotions. Taken together, these results lend support the motor origin hypothesis for the musical expression of emotions.

## Introduction

Musicians can play the same music score using different expressive strategies and emotional intentions. Several studies have investigated the encoding and perception of this emotional expressivity in music performance (e.g. [1]–[5]; see [6] for a review). Typically, the same score was played with different emotional intentions and listeners recognition capabilities were assessed via either categorization, or rating tasks. Using this methodology, properties of the music associated with different emotional categories were highlighted, both for performance and perception.

A relevant question then concerns the origin of the ability to communicate emotions in music performance. Naturally, here a musician makes use of knowledge acquired during his/her musical training. Studies have shown that expressive skills are regarded as highly important [7], and that both practice and experience improve the performer's ability to convey expressive intentions and the listener's ability to recognize them [8]. However, it is still unclear whether these expressive rules are a mere music-performance convention learnt through musical training and listening, or instead whether their acquisition relies on knowledge drawn from a non-music domain. Similarly, what is the origin of the perceptual criteria used by a listener to recognize emotional expressions in music performance?

We would like to suggest that both the expression and recognition of emotion in music draw from knowledge about the sounds of expressive body movements, an explanation we refer to as the motor origin hypothesis for the musical expression of emotions. We attempted to test this hypothesis by drawing parallels between the musical expression of emotions and the expression of emotions with a non-musical everyday motor activity such as walking.

### Expression and recognition of emotions

According to his componential patterning theory of affective expression, Scherer [9] describes the emotion-specific expressive vocal patterns as outcomes of stimulus evaluation checks (multi-level appraisals) that result in specific adaptive behaviors. These behavioral responses are accompanied by supportive activations in the somatic and autonomic nervous system (e.g. a increase in general muscle tension preparing for a fight or flight response) that are in turn also thought to influence the expressive sound production in the vocal tract, a mechanism Scherer termed push effects (as opposed to pull effects which are culture-specific learned expressive cues). As these responses also affect other parts of the human motor system, emotional expressions are manifested in body movements that also lead to corresponding emotion recognitions [10], [11]. The recent trend in cognitive psychology to focus on body-mind interactions [12], [13] has also lead to stronger research focus on the embodiment of emotional processes. De Gelder [11], for example gives a detailed account of emotional body language, comparing emotional communication in several modalities, showing that the body plays an underestimated role in emotion recognition.

### The Motor Origin Hypothesis of Emotional Expression in Music

Already 19th century musicologist von Hausegger [14], described musical emotions as resulting from the internal mimicking of the expressive movements that were necessary to produce them (for a more recent account see also [15]). Furthermore, Davies [16] argued that music might be perceived as emotional based on the similarities to other human expressions; an idea that was later also termed *resemblance theory* [17]. Accordingly, Juslin and Laukka [6] reviewed literature concerning the acoustical expression of emotions in the music performance and vocal domains and reported that strong similarities emerged. In particular, independently of whether an emotional intention is expressed through instrumental music, singing voice, or speech, the same emotions are associated with similar variations in acoustical properties such as average tempo, articulation, or loudness. These similarities were interpreted as substantiating a vocal-origin hypothesis according to which the ability to express emotions expression in the music–performance domain relies on the imitation of expression of emotions in the vocal domain.

Importantly, Juslin and Laukka discard an alternative explanation for the observed similarities between the two domains: emotions are expressed in similar ways in music and vocal performance because they both result from a motor activity that expresses emotions in coherent ways independent of the particular effector. We refer to this hypothesis as the motor-origin hypothesis (see also [13]). Hearing action sounds can lead to an understanding of associated actions through activations of mirror neurons [18]. This coupling is thought to be based on Hebbian learning that may even have the capacity to bind perceptions, actions, and also emotional expressions together [19]. Music performance learning is often used as a model to study this perception-action cycle, illustrating the complex interactions between motor control and auditory perception in music [20]–[22].

According to Juslin and Laukka, whereas the vocal-origin hypothesis would require the transmission of an expressive code within a single modality – audition – the motor-origin hypothesis would instead require a learning mechanism of crossmodal nature, which translates between the auditory and motor domains. It would follow that the vocal-origin hypothesis explains the music-vocal similarities in a more economical way, since it would not require a translation of expressive codes. Two points can however be objected to these conclusions. Firstly, crossmodal consistencies are evaluated on a daily basis by our perceptual systems, since the very first days of our life [23]. Secondly, a motor-to-auditory translation of the expressive code would not be strictly necessary since motor activity frequently generates acoustical signals (e.g., walking sounds, [24]). We therefore think it is plausible to hypothesize a common motor origin for the expression of emotions in all those domains of human activity that result in the generation of an acoustical signal.

### Walking and music performance

Why should walking provide an appropriate framework that allows testing the motor-origin hypothesis of emotional expression in music? Music performance and walking share several similarities like the fact that they represent common motor activities that lead to audible sounds. For music, this is mostly the main intention of the executor, for walking, those sounds are rather a by-product. That walking could be a good vehicle for the expression in music is supported by at least two empirical studies.

The first one investigated the acoustical structure of walking sounds outlining features known to differentiate between emotional playing styles in music [25]. In this study several similarities between articulation in piano playing and walking or running were drawn, where the amount of temporal overlap among adjacent tones/footstep sounds distinguished between staccato and legato articulation. It was found that the overlap time in both walking and legato articulation increases linearly with decreasing pace/tempo, and that the time-in-the-air between feet (running) and between fingers (staccato) increases with decreasing pace/tempo following the same linear law.

Another clear example of the connection between music performance and body motion is that of the final ritardandi. The mean body velocity of stopping runners was found to be very similar with the mean tempo pattern of final ritardandi in music performances, and this property was confirmed also in listening tests [26]. Thus, musical expressions of emotion may show similarities to walking.

Emotion recognition operates very fast so that decoding of expressed emotion in music can result from very short musical stimuli, less than 1 s [27], [28]. This confirms that structural musical elements that are dependent on larger time windows (like tonal tension, metric hierarchy, etc.) are not mandatory in order to express or perceive emotion in music. Thus, recognizing emotional expressions in music might be at least partially based on the same mechanism that is also employed when emotions are recognized in the short and hierarchically unstructured walking sounds.

### Aims and Experimental Design

In this study, we tested the motor-origin hypothesis for the emotional expression in music by comparing the use of expressive sound features in music to an everyday motor activity, walking. Two experiments were carried out in order to test for hypothesized similarities between emotion expression and recognition in music and in walking.

The first experiment focused on the production of emotionally expressive walking sounds. To rule out any confounding effects, musically untrained individuals participated in this experiment. They were asked to walk as if feeling one of several different emotions. The acoustical features of the walking sounds were extracted, and compared with those characterizing emotionally expressive musical performances [6]. It was hypothesized that emotional intentions in walking modulated several acoustical features in the same way as in music performance. A confirmation was interpreted as supporting a common origin for the expression of emotions in music and non-musical motor activity at large.

Subsequently, a second experiment investigated the ability to recognize the emotional intention of a walker when only acoustical information is available. It was hypothesized that the majority of the listeners had higher-than-chance performance in the perception of different emotions in walking sounds. It was also tested if music expertise moderated emotion recognition performance, because we argued that listeners did not use music-related knowledge to recognize emotions in walking sounds. Recognition of the emotions of a walker was also compared with performance in the recognition of emotion-independent properties of the walking events. Finally, we investigated the acoustical parameters used by listeners to recognize the emotions and the emotion-independent properties of the walking event.

## Experiment 1

### Method

#### Ethics Statement

Prior to participating in both experiments, individuals were informed of their general goals and of the procedures involved. They gave oral consent to participating in the study. At the time the experiments were conducted, no ethics approval was required from the KTH for behavioral studies such as those reported in this manuscript. The local Ethics board at the KTH subsequently approved highly similar experiments conducted in the same Institute one year later the experiment reported in this manuscript was conducted. Neither of the experiments involved deception or stressful procedures. Participants were informed that they were free to leave the experiment at any time, and that their data would have been treated anonymously. The research reported in this manuscript was carried out according to the principles expressed in the Declaration of Helsinki. Participants in both experiments were recruited on a voluntary basis from the students and staff of the KTH. They were compensated for their participation with cinema tickets.

#### Participants

Seven walkers took part in the experiment on a voluntary basis (age: 28–62 years, M = 38 years; 2 females). All the walkers had never received musical training. Table 1 reports anthropometric measures, age, gender and shoe properties for each of the walkers. Sole hardness was measured on an ordinal scale.

**Table 1:** Participant–specific descriptors for Experiment 1.

Walker	Age	Gender	Weight (in kg)	Height (in cm)	Sole length (in cm)	Sole width (in cm)	Sole material	Sole hardness
1	32	Male	85.5	192	32	15	Leather	2
2	62	Female	70	172	28	8.5	Wood	4
3	28	Male	73	179	29.5	11.5	Hard rubber	3
4	41	Male	100	192	28	10	Wood	4
5	34	Male	72	189	29.5	11.5	Hard rubber	3
6	36	Male	92	167	30	11.5	Soft rubber	1
7	32	Female	58	181	27	9.5	Wood	4

#### Procedure and apparatus

Participants walked along a 10 m long track, marked on a linoleum floor with scotch tape. They wore a pair of their own shoes, selected so that they could make it possible to easily generate relatively loud walking sounds. They were instructed to walk as if either feeling a certain emotion, or as if in a “neutral”, i.e. emotionless mood. Four basic emotions were investigated: happiness, sadness, anger and fear. Participants practiced each of the walking styles three times before the recording session was started. No feedback concerning the appropriateness of the adopted walking styles was given. The five walking styles were performed in blocked-randomized order for each of five repetitions, for a total of 25 trials. Sounds were recorded with a Brüel & Kjær DPA type 4021 microphone, connected to an USB Tascam US-122 soundcard (44,100 Hz sampling rate, 16-bit resolution). The microphone was placed 1 m above the floor level on the side of the middle of the walking path.

#### Acoustical features

The analysis of the acoustical properties of the walking sounds comprised three subsequent stages. Firstly, those portions of the sound connected to the initiation and termination of the locomotive behavior were eliminated, so as to avoid their influence on the values of the acoustical parameters extracted to investigate the effects of the emotional intention. The extracted sounds were subsequently processed so as to identify those portions of the waveform generated by the impact of the shoe sole on the floor. Finally, a large set of features was extracted to characterize the acoustical structure of the walking events.

The amplitude envelope of a walking sound was extracted from the Hilbert transform of the waveform [29]. Hearing-range amplitude fluctuations were attenuated by forward-reverse filtering of the amplitude envelope using a third-order Butterworth filter with a low-pass cutoff frequency of 16 Hz [30]. Walking-sound amplitude increased and decreased as the walker approached and moved away from the microphone, respectively, peaking approximately at the center of the sequence. Also, pace, the temporal distance between subsequent footsteps, accelerated and decelerated around the beginning and end of the sequence, respectively. Analyses focused on the central portion of the recordings, in order to minimize the effects of walker-to-microphone distance and of the initiation and termination of the locomotion on the values of the acoustical descriptors. A maximum of seven subsequent footstep sounds was therefore isolated from each of the recorded walking sounds samples. They had a peak level 10 dB higher than the average level of the entire sound, as estimated from the amplitude envelope. The central footstep sound had maximum within-sequence level, i.e., it had likely been generated closest to the microphone.

**Figure 1:**
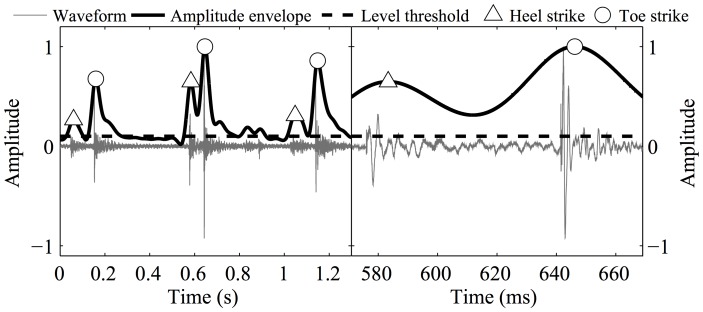
Identification of amplitude envelope peaks generated by heel and toe strikes. The right figure details the lag of the amplitude envelope relative to the waveform. The amplitude envelope and the level threshold have been scaled so that the maximum envelope level matches the maximum waveform amplitude.

Each of the footstep sounds could potentially include two subsequent impulsive signals, generated by the sequential heel-toe strike (see Fig. 1). In practice, however, a footstep sound could also comprise one single impulsive signal, either resulting from the simultaneous impact of the heel and toe on the floor, or from a low impact force of either the heel or the toe. Notably, walking sounds could also contain the sounds of the friction of the walkers' clothes.

Local amplitude peaks most likely generated by heel or toe strikes were identified on the basis of an analysis of the amplitude envelope. Envelope peaks whose level was not higher than 10 dB relative to the background noise level were discarded because they likely had a different origin than the impact of the sole on the floor. Envelopes that included a peak higher than 10 dB from the background noise, but whose duration was shorter than 20 ms were also discarded because previous acoustical studies of walking sounds revealed that the duration of both heel- nor toe-strike impact sounds did not exceed 40 ms [31]. The rate of level increase for the initial portion of the isolated envelopes was finally computed in order to differentiate between impulsive signal such as those generated by a sole-to-floor impact, characterized by a fast level rise from sound onset, and non-impulsive sounds, such as those generated by the friction between the clothes of the walkers, characterized by a slower level increase. Linear regression was used to compute level rise rate from the 20-ms envelope portion ending at the peak. Peaks associated with a level rise rate of less than 100 dB/s were discarded.

Once the footstep sound amplitude envelope peaks were isolated, a decision was made about which of them had been generated by a heel strike or by a toe strike, and about which footstep sound contained only one impact. This final decision was carried out on the basis of the following assumptions: the minimum temporal distance between temporally adjacent footsteps is 250 ms [32], [33]; heel strikes always precedes a toe strike; the maximum temporal distance between heel and toe strikes is 150 ms (Pastore et al. [31], found an average heel-to-toe strikes distance of 112 ms). If the above criteria isolated one single amplitude peak per footstep, this was taken as originating from the simultaneous impact of the heel and toe on the floor. Fig. 1 details this analysis. The measurement of the acoustical structure of the walking sounds comprised two steps. Initially, a set of 10 time-varying features measured the variation of acoustical structure throughout the walking-sound sequence. Subsequently, summary statistics were adopted to reduce the time-varying features to one single number that characterized the acoustical structure of the entire walking excerpt. Six time-varying acoustical features were extracted from the amplitude envelope. Firstly, the level of heel and toe strike peaks, H.lev and T.lev, respectively, measured in dB relative to the background noise threshold dB_thr_. Secondly, the time interval between subsequent toe strikes, T2T, and between heel and subsequent toe strikes, H2T, as measured from the temporal location of the envelope peaks. Thirdly, the duration of footstep sounds F.dur, given by the time interval between the first above-threshold envelope sample before the heel peak, and the last above-threshold sample after the toe peak. Finally, Art, a walking-performance measure inspired by the articulation descriptor used in music performance, here intended as legato and staccato. In piano performance, two notes are played legato when the second-note key is pressed while the first-note key is still pressed, whereas two notes are played staccato when the second-note key is pressed when the first-note key has been released already at a time corresponding to about 50% of the time interval between the two notes. Here, the Art measure was defined as the ratio of T2T to the duration of silence separating subsequent toe and heel strikes. To this purpose, the duration of the silence between subsequent toe and heel strikes was defined as the time interval between the last above-threshold envelope sample after a toe strike, and the first above-threshold sample before the subsequent heel strike. Similarly to articulation measures in music performance studies, low values of Art correspond to a more “staccato” walking sound and high Art values correspond to a more “legato” walking style [25]. Four time-varying descriptors characterized the spectrum of the footstep sounds. They were extracted from a fast Fourier transform of 2048 samples of the waveform (Hanning window; window length  = 46 ms), starting 10 ms before the heel and toe amplitude envelope peaks. Spectral descriptors were computed considering frequencies in the 16–16000 Hz range. The first two descriptors measured the spectral center of gravity SCG, i.e., the amplitude-weighted average spectral frequency (H.SCG and T.SCG for heel and toe strikes, respectively). The second two descriptors measured the spectral mode, i.e., the frequency of the most intense spectral component (H.mod and T.mod for heel and toe strikes, respectively).

Two measures were extracted from each of the ten time-varying descriptors: the average value and the standard deviation within the walking-sound sequence, indicated by the subscripts mea and std, respectively. For instance, T.lev_mea_ measured the average of the toe strike peak levels and H.mod_std_ measured the standard deviation of the spectral mode of the heel-strike-impact sounds. In total, 20 descriptors were computed for each of the footstep sounds.

#### Data Analyses

We carried out a walker-by-walker analysis of the similarity between the acoustical expression of emotions in walking and musical performance. Juslin and Laukka [6] report a summary of the previously published works on the acoustics of the expression of emotions in music performance. Specifically, several acoustical properties are listed (e.g., tempo), along with the number of music performance studies where a given emotional intention was associated with a specific value for each of the acoustical features (e.g., 20/20 studies report a fast tempo for anger; 9/10 studies report a fast tempo for fear; 23/28 studies report a fast tempo for happiness; 23/23 studies report a slow tempo for sadness). Among the acoustical features listed in Juslin and Laukka, we selected those that could be measured in the walking sounds: tempo, sound level average and variability, high-frequency energy, and articulation average and variability. Music-performance features derived from properties likely constant within the walking-sound sequence signals were not considered (e.g., pitch contour or attack time). The musical descriptor timing variability was also considered. In the walking domain it was defined as the tempo variability within the walking-sequence and measured the extent to which pace (“beat”) varied with reference to the “nominal” value of the average within-walking sequence pace.

We tested for higher-than-chance similarities in the acoustical expression of emotions between the music and walking performance domains. We used the procedure described in McKean, Naranjo, and Huitema [34] to test rank-order hypotheses about the ordering of the values of the acoustical features of the walking sounds as a function of the emotional intention. In particular, for each walker, the similarity between the acoustical expression of emotions in music and walking performance was defined as the Spearman rank correlation between the emotion-specific ranks of acoustical properties in the two domains. Correlations of 1, 0 and -1 indicated perfect similarity, no similarity and perfect dissimilarity, respectively. For each walker, and for each of the considered acoustical features, we used the bootstrap approach (BCa, [35]; 10,000 bootstrap samples for each test) to test for higher-than-zero similarities between the music performance and walking domains.

### Results and Discussion

Table 2 pairs the musical features from Juslin and Laukka [6] with those extracted from the walking sounds. For each of the selected acoustical features, and for each of the emotional intentions investigated in this study, Table 2 shows grand-average value observed in the database of walking sounds, and the most frequent value emerging from the review of music performance studies by Juslin and Laukka.

**Table 2:** Acoustical features as used in expression of emotions in music performance, adapted from Juslin and Laukka (2003), and across–walkers median value of the corresponding acoustical features computed from the recorded walking sounds.

Music performance					Walking performance				
Acoustical feature	Emotion				Acoustical feature	Emotion			
	A	F	H	S		A	F	H	S
Tempo	Fast	Fast	Fast	Slow	T2T_mea_ (ms)	509	684.7	514.6	714.9
Timing var.	Medium	Large	Small	Med./Lar.	T2T_std_ (ms)	28.6	65	50.8	92.3
SL	High	Low	Medium	Low	H.lev_mea_ (dBthr)	18.8	10.7	15.5	11.3
					T.lev_mea_ (dBthr)	18.3	10.6	14.8	9.7
SL var.	High	High	High	Low	H.lev_std_ (dBthr)	4.2	4.4	4.2	3.7
					T.lev_std_ (dBthr)	3.8	3.6	3.9	3.9
High fr. en.	High	Low	Medium	Low	H.SCG_mea_ (Hz)	3221	3808.6	3171.7	3530
					T.SCG_mea_ (Hz)	3113.3	3851	3314.9	3793.5
Art.	Staccato	Stac-cato	Staccato	Lega-to	Art_mea_	0.4	0.7	0.5	0.8
Art. var.	Medium	Large	Large	Small	Art_std_	0.2	0.1	0.1	0.1

*Note. A =  Anger; F =  Fear; H =  Happiness; S =  Sadness; SL =  Sound Level; var. =  variability; High fr. en. =  High frequency energy; Art. =  Articulation; Med./Lar. =  Medium/Large; H =  Heel; T =  Toe; lev =  level; SCG =  Spectral Center of Gravity; mea =  mean; std =  standard deviation; dBthr =  dB from threshold level of the background noise.*

**Figure 2:**
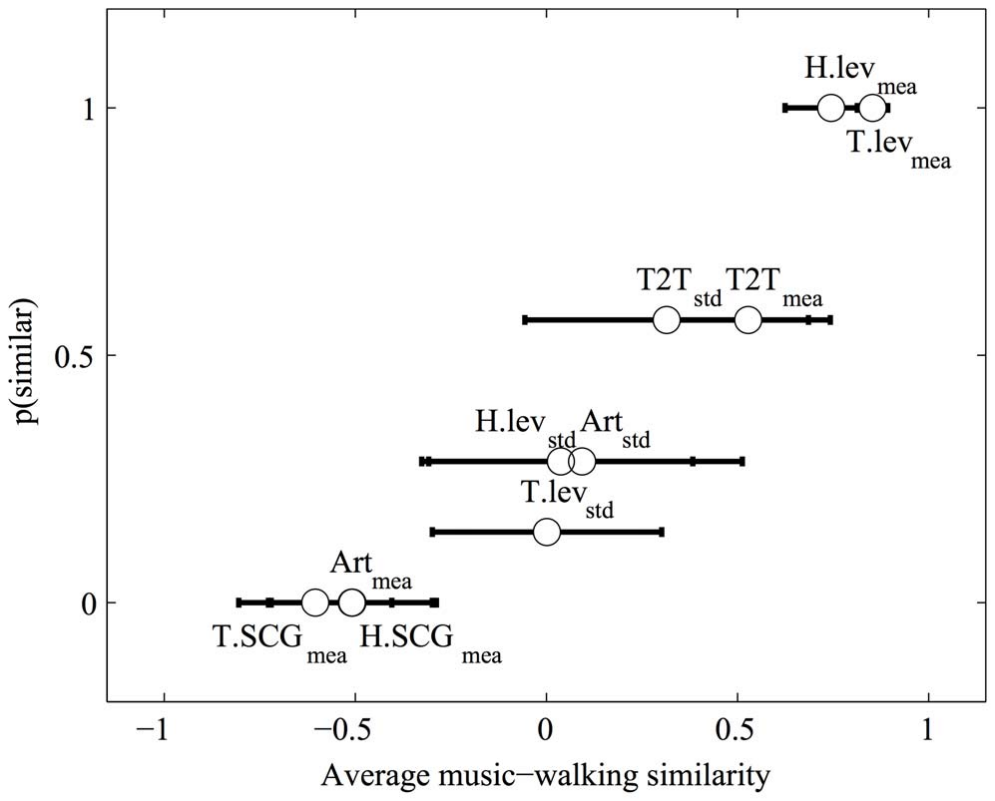
Similarity in the acoustical expression of emotions in music and walking performance. The abscissa shows the similarity scores averaged across walkers (Spearman rank correlation coefficients). The ordinate shows the proportion of walkers for which a higher-than-chance similarity is observed. H = Heel; T = Toe; lev  =  level; SCG  =  Spectral Center of Gravity; Art  =  articulation; mea  =  mean; std  =  standard deviation. Error-bars  =  95% confidence intervals for the average performance.

Rank-order hypotheses were derived from this literature review on the acoustical expression of emotions in music performance (e.g., in music performance the rank-order of expressive emotions relative to sound level defines the following hypothesis in the walking domain: fear  =  sadness < happiness < anger). The results of this analysis are summarized Fig. 2 (see also S1 Table). Across participants, heel and toe amplitude level means show the highest levels of music/walking similarity followed by T2T mean and standard deviations, indicating a similar usage of these acoustical features for the expression of emotions in the two domains. Articulation and high frequency measures (SCG) did not show any similarity to the predicted expressive patterns. Sound intensity, regularity and tempo of walking sounds showed a similarity in cue usage across the two domains. It is likely that participants did not control actively the spectral centroid and the articulation features leading to a lack of similarities concerning these features. For example, a better control of spectral centroid would have been possible if participants were able to manipulate the sole and floor materials, whereas a batter control of articulation would have been possible if participants were able to actively manipulate the duration of the impact sounds they generated when walking, a hard task given the strong constraints imposed by the mechanics of the walking sound source.

Overall, the similarities observed between the acoustics of emotional expression in music and walking performance with the musically-untrained participants in this experiment are consistent with the motor hypothesis for the origin of emotional expressivity in music. Notably, however, the observation of such similarities only partially supports the motor-origin hypothesis because it doesn't prove that the acoustical patterns for the expression of emotions in non-music motor performance can also be picked up by a perceiver.

## Experiment 2

In Experiment 2, we investigated the ability to recognize emotions from the acoustical information carried by the walking sounds recorded in Experiment 1. An absence of significant associations between the perception of emotions in walking sounds and musical expertise was interpreted as disconfirming the hypothesis according to which listeners make use of music-related knowledge when recognizing emotions in walking sounds. We finally tested on which acoustical parameters listeners based their recognition ratings and compared them to the parameters used for encoding of emotion in walking sounds.

### Methods

#### Participants

Fourteen listeners took part in the experiment on a voluntary basis (age: 25–59 years, M = 31 years; 7 females). All of them declared normal hearing. Their mean number of weekly hours of musical listening was 2 (SD = 2.1); the mean number of years of music performance training was 11.5 (SD = 8.7); 8 out of the 14 participants were currently practicing an instrument.

#### Stimuli and apparatus

We selected one walking excerpt among the five repetitions of each of the five walking styles (angry, happy, fearful, normal and sad), for each of the seven walkers that participated in Experiment 1, for a total of 35 stimuli. Stimuli were stored on the hard disk of an Intel-PC workstation, equipped with a Sound Blaster Live sound card. Audio signals were presented through AKG K240 headphones, connected to the sound card output. Participants sat inside an acoustically isolated room. The peak levels of the signals ranged from 32.7 to 62.2 dB SPL, as measured with an Ono Sokki LA-210 sound-level meter.

#### Procedure

We adopted the Verbal Attribute Magnitude Estimation (VAME) technique [36] to investigate the recognition of the intended walking emotion, and of several emotion-independent properties of the walking sound source. Accordingly, stimuli were rated by moving a slider along 13 bi-polar scales, each defined by a different sentence and by it's negation (e.g., “the walker is sad” and “the walker is not sad”). Five of the rating scales were related to the walkers' emotions: “the walker is angry”; “the walker is fearful”; “the walker is happy”; “the walker is normal”, i.e., emotionless; “the walker is sad”. Four of the scales referred to emotion-independent properties of the walker: “the walker is female”; “the walker is male”; “the walker is light”; “the walker is heavy”. Four of the scales referred to the properties of the shoe soles: “the sole is large”; “the sole is small”; “the sole is hard”; “the sole is soft”. For each of the trials, the participant was presented with one sound stimulus and the 13 rating scales, each comprising a horizontal slider, and arranged randomly along the height of the screen. The trial ended after the stimulus was rated along each of the 13 scales. Participants could replay the stimulus as many times as needed. Before the beginning of the experimental phase, they practiced the rating procedure with four walking excerpts, not included in the main experiment. After this training phase, participants were presented at least once with all of the experimental stimuli in random sequential order. Each of the stimuli was rated once by each of the participants, in random order, for a total of 35 trials. The experiment lasted approximately one hour.

#### Data analyses

Four different analyses were carried out. Firstly, we analyzed participants' consistency in the use of scales defined by opposite adjectives (e.g., female vs. male). Data of inconsistent participants were discarded. Secondly, performance in the recognition of each of the investigated attributes of the walking event was quantified. Thirdly, effects of musical expertise on recognition performance were tested. Finally, we analyzed the acoustical correlates of participants' ratings. All analyses focused on rank information, so as to avoid the assumption concerning the shape of the monotone transforms relating variables of interest.

For each of the participants, we quantified the consistency between the ratings along scales defined by opposite emotion-independent attributes of the walking event, i.e., female walker vs. male walker; heavy walker vs. light walker; hard sole vs. soft sole; large sole vs. small sole. The Spearman rank correlation between ratings along opposite scales was used to this purpose. Consistent participants were expected to give diametrically opposite ratings along opposite scales. As such, for each of the participants, we tested whether the Spearman rank correlation coefficient between ratings along each of the pairs of opposite scales was significantly lower than zero. For one of the participants, two of the opposite-scales correlations were not significantly lower than zero (Spearman rank correlation coefficient ≥−0.27; *p*≥0.06; df = 33). The data of this participant were not further considered. For all the other participants, strong negative correlations were observed between ratings along opposite scales (grand-average Spearman rank correlation coefficient  = −0.82; SD = 0.11; *p*≤0.006; df = 33). Ratings given along opposite scales were reduced to a single variable by means of Principal Components Analysis (PCA). In particular, for each of the participants, and for each pair of opposite-scale ratings, we conducted a PCA based on the ranks of the reduced variables. For each pair of opposite-scale ratings, the first PC was retained as reducing variable, which was always strongly correlated with the reduced variables (grand-average of the absolute Spearman rank correlation coefficient between reduced and reducing variables across all participants: M = 0.91; SD = 0.06). Following this methodology, the “female” and “male” ratings were converted into a “maleness” scale; the “light” and “heavy” ratings into a “heaviness” or “walker weight” scale; the “small” and “large” ratings into a “largeness” or “sole size” scale; the “soft” and “hard” ratings into a “sole hardness” scale.

We finally investigated the acoustical criteria for the recognition of the properties of the walking events, by focusing on ratings averaged across participants. This analysis involved two sequential steps. Firstly, a data-reduction step that aimed at merging together groups of strongly correlated acoustical descriptors, and groups of strongly correlated rating scales. Secondly, a regression analysis, aiming at assessing significant associations between ratings and features of the walking sounds.

A Principal Component Analysis (PCA) was carried out on the ranks of the groups of strongly correlated acoustical descriptors [37], and the first Principal Component (PC) was retained as the final reducing variable. This data-reduction step was guided by means of a hierarchical cluster analysis (average linkage) of the between-descriptors correlation, carried out on a measure of the distance between acoustical descriptors, defined as 1 minus the absolute value of the between-descriptor correlation. More specifically, starting from the condition where each of the descriptors was in an isolated cluster, each of the clusters of descriptors was independently reduced to a single PC, and the correlation between the PCs derived for of the different clusters was computed. If none of the absolute between-cluster correlations exceeded a threshold of 0.50 the procedure was terminated. Otherwise, the number of clusters of descriptors was reduced by one and the procedure was iterated. This procedure generated in output six PCs of acoustical descriptors (see Table 3). The six PCs summarized well the acoustical variables they reduced (average absolute Spearman rank correlation between acoustical variables and reducing PC = 0.86, SD = 0.08).

**Table 3:** Spearman rank correlation coefficients between acoustical features and Principal Components (PCs).

Acoustical feature	PC	Rank correlation with PC
H2T_mea_	1	.93
H2T_std_	1	.92
Art_mea_	2	.91
Art_std_	2	−.80
F.dur_mea_	2	−.90
F.dur_std_	2	−.78
H.lev_std_	2	−.83
T.lev_std_	2	−.72
T2T_mea_	2	.69
H.SCG_mea_	3	−.91
H.lev_mea_	3	.90
T.SCG_mea_	3	−.89
T.lev_mea_	3	.89
T2T_std_	4	1.00
H.SCG_std_	5	.89
T.SCG_std_	5	.87
H.mod_mea_	6	.88
H.mod_std_	6	.90
T.mod_mea_	6	.76
T.mod_std_	6	.84

Strong Spearman rank correlations were present also between the ratings given along the different response scales, averaged across participants (see Table 4). Groups of strongly correlated rating scales were reduced to a single variable using the same procedure adopted for the acoustical descriptors. Three PCs of rating scales were extracted. They were weakly correlated with each other (average absolute Spearman rank correlation coefficient between PCs = 0.23; SD = 0.13), and accounted well for the reduced rating scales (average absolute Spearman rank correlation coefficient between rating scales and respective reducing PC = 0.90; SD = 0.09; see Table 4). The first PC reduced ratings along the “Angry” and “Sad” scales. It increased in value with increasing “Angry” ratings and decreasing “Sad” ratings. With reference to Russell's circumplex model [38], the emotions captured by these two scales differ vastly along an arousal dimension, and little along a valence dimension. The first PC was therefore termed Arousal. The second PC reduced ratings along the “Happy”, “Normal” and “Fearful” scales. It increased in value with increasing ratings along the “Happy” scale, and with decreasing ratings along the “Normal” and “Fearful” scales. Focusing on the happy and fearful emotions, and on their large difference in valence, as compared to their difference in arousal, the second PC was termed Valence. Finally, the third PC reduced ratings along all the remaining scales, referred to the emotion-independent properties of the walking event, and increased in value for increasing ratings along the “Female”, “Light”, “Small” and “Hard” scales, and for decreasing ratings along the “Male”, “Heavy”, “Large”, and “Soft” scales. The third PC was termed Gender.

**Table 4:** Experiment 2. Spearman rank correlation coefficients between data from the different rating scales, averaged across listeners.

Scale	An	Ha	Fe	No	Sa	Fm	Ma	Li	He	Sm	La	Ha	Soft
Angry	–	.01	.36^*^	.06	−.77^***^	.44^**^	−.39^*^	.26	−.16	.29	−.24	.27	−.29
Happy		–	−.35^*^	−.72^***^	.13	.21	−.23	.31	−.34^*^	.36^*^	−.33^*^	.04	0
Fearful			–	.66^***^	−.62^***^	.17	−.19	.18	−.15	.11	−.09	−.05	.06
Normal				–	−.25	−.05	.05	−.07	.11	−.17	.14	−.03	.01
Sad					–	−.41^*^	.43^*^	−.22	.15	−.24	.22	−.25	.23
Female						–	−.98^***^	.91^***^	−.85^***^	.92^***^	−.91^***^	.82^***^	−.74^***^
Male							–	−.90^***^	.85^***^	−.92^***^	.92^***^	−.81^***^	.72^***^
Light								–	−.95^***^	.96^***^	−.95^***^	.65^***^	−.59^***^
Heavy									–	−.93^***^	.92^***^	−.56^***^	.49^**^
Small										–	−.96^***^	.65^***^	−.59^***^
Large											–	−.70^***^	.62^***^
Hard												–	−.96^***^
PC	1	2	2	2	1	3	3	3	3	3	3	3	3
Corr.	.94	.81	−.75	−.95	−.93	.97	−.96	.96	−.9	.95	−.96	.81	−.75

Note. PC  =  Principal Component; Corr. =  rank correlation coefficient. * p<0.05; **p<0.01; *** p<0.001. For all the correlations between rating scales and the respective PC: p<0.001.

### Results and Discussion

Performance in the recognition of the investigated properties of the walking event was quantified on a participant-by-participant basis. To this purpose, we adopted the same methodology used to test for significant similarities between the expression of emotions in the music- and walking-performance domains (Experiment 1). In particular, recognition performance was defined as the Spearman rank correlation coefficient between the ratings on a particular scale, and the value of the related property of the walking event. For example, performance in the recognition of the weight of a walker was defined as the Spearman rank correlation between the ratings along the “walker heaviness” scale and the actual heaviness of the walker. Within this context, a correlation of 1 and 0 indicated perfect and chance-recognition performance, respectively, whereas a correlation of -1 corresponded to perfect misidentification (e.g., the listener systematically assigned lower heaviness ratings to heavier walkers). Following the procedure detailed in McKean et al. [34], the bootstrap approach [35] was used to test for higher-than-chance recognition performance, i.e., higher-than-zero Spearman rank correlation between ratings and related actual properties of the walking event (N bootstrap samples  = 10,000). Shoe sole size recognition performance was assessed with reference to the product of the sole length and width (Table 1).

**Figure 3:**
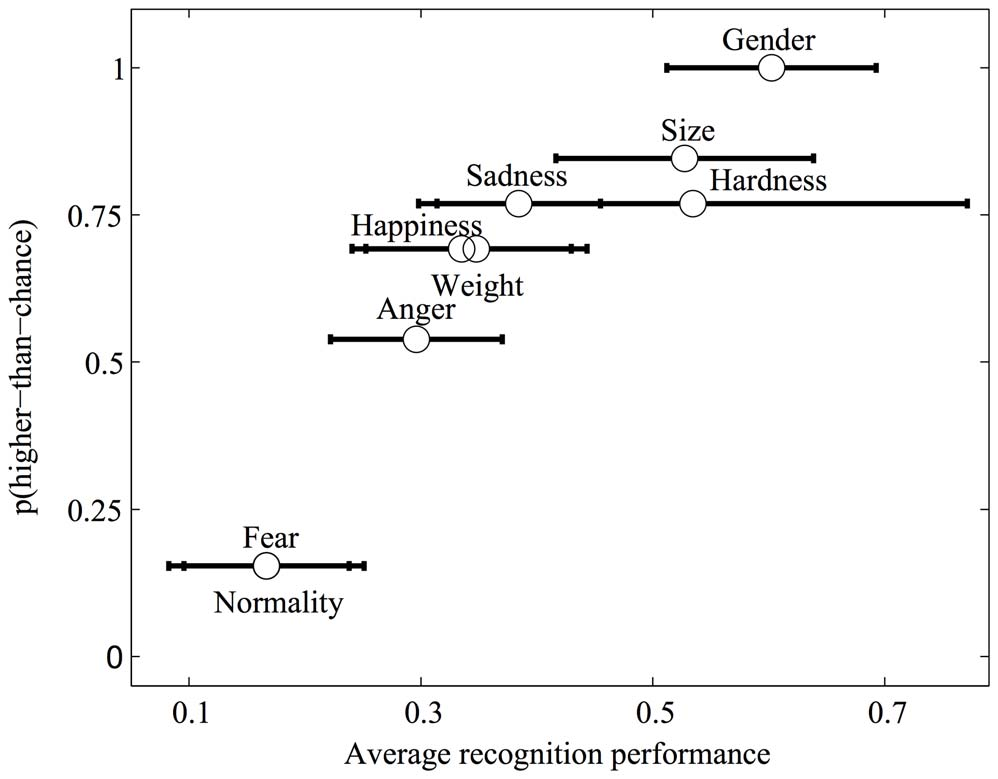
Analysis of the performance in the recognition of the investigated properties of the walking event. Performance scores of 1, 0 and -1 correspond to perfect recognition, chance recognition and perfect mis-recognition, respectively. The abscissa shows, for each of the properties of the walking event, performance scores averaged across all the listeners (Spearman rank correlation coefficients). The ordinate shows the proportion of listeners with higher–than–chance recognition performance for each of the estimated walkers' properties. Error-bars  = 95% confidence intervals for the average performance. Gender  =  “maleness”.

In the case of binary properties (e.g., gender), the actual property of the walking event was coded as a binary variable (e.g., male  = 1; female  = 0 to test recognition performance with the “maleness” ratings). We tested separately recognition performance for each of the intended walking emotions, including the “normal” walking style. To this purpose, the ratings were correlated with binary variables coding for whether a given walking sound had been generated with a target emotional intention or not. For example, “sad walker” ratings were correlated with a binary variable coding for whether the actual emotional intention was sad (value  = 1) or not (value  = 0). The results of this analysis are summarized in Fig. 3 (see also S2 Table). The highest recognition performance was observed for the non-emotional attributes gender, sole size and sole hardness. Importantly, performance in the recognition of the three basic emotions sadness, happiness and anger was significantly higher than chance for at least half of the participants. Overall, listeners showed good abilities to recognize both emotion-and non-emotion properties of the walking events, exception done for the normal and fearful walking styles, which were recognized significantly better than chance by only two participants.

We subsequently tested whether measures of the musical expertise of the participants were associated with their ability to recognize the emotions of a walker. Three music-expertise measures were considered: the number of weekly hours of musical listening; the number of years of music performance training; their current status as music performer (currently not practicing  = 0; practicing  = 1). We computed the Spearman rank correlation coefficient between each of the three measures of musical expertise on the one hand, and the each of the five performance scores for the recognition of each of the walkers' emotion (“normal” included), on the other, for a total of 15 correlations. We were interested in assessing whether participants with higher levels of musical expertise were better at recognizing the emotional walking intention, and thus tested the unidirectional hypothesis Spearman rank correlation coefficient >0. Only two of the 15 correlation coefficients were significantly higher than zero: that between performance in the recognition of anger and the hours of weekly musical listening (Spearman rank correlation coefficient  = 0.61, *p* = 0.01; df = 11), and that between the performance in the recognition of normal, i.e., emotionless walking, and the current status as performer (Spearman rank correlation coefficient  = 0.66; *p*<0.01; df = 11). The latter might be explained by the higher ability of musicians to detect small variations compared to nonmusicians [39], and therefore since musicians usually play with expression, they are more trained to recognize a lack of expression in a sequence of sounds. Most importantly, all of the other 13 correlations were not significant (average absolute Spearman rank correlation coefficient  = 0.20; SD = 0.13; *p*≥0.09; df = 11). Therefore, the most frequent trend emerging from this analysis is that musical expertise was not associated with a significant increase in the ability to recognize the emotions portrayed by the footstep sounds.

For each of the three PCs of rating scales, we built a multiple rank regression model [40] using the six PCs of acoustical descriptors as predictors (Table 5). For each of the multiple regression models, predictors were selected based on the stepwise method. Finally, the predictive power of the different predictors was compared based on a measure of the size of the effect of the acoustical PCs onto the rating PCs, the partial *R* ^2^.

**Table 5:** Experiment 2. Summary of multiple regression analysis of population ratings.

Acoustical PC	b	SE of b	*β*	partial R^2^
Arousal PC (*df* = 31)				
2	−0.36	0.14	−3.66*	0.18
3	0.37	0.14	3.77*	0.19
4	−0.37	0.12	−3.81**	0.23
Valence PC (*df* = 33)				
4	0.38	0.16	3.85*	0.14
Gender PC (*df* = 32)				
1	−0.32	0.14	−3.21*	0.14
6	0.53	0.14	5.4***	0.32

Note. PC  =  Principal Component; * p<0.05;**p<0.01; *** p<0.001.

The final model for the Arousal PC of rating scales included three PCs of acoustical descriptors, the second, third and fourth, and explained 55.54% of the variance of the ranks of the Arousal PC. The third PC includes the level variables H.lev_mea_ and T.lev_mea_; the fourth PC includes the timing variable T2T_std_, which all showed significant similarities between the walking and music performance domains in at least 50% of the participants in Experiment 1 (see Fig. 2).

The final model for the Valence PC included only the fourth PC, and explained a mere 14.15% of the variance of the ranks of the Valence PC. This fourth PC included only the timing variable T2T_std_ that in Experiment 1 also showed significant similarities between walking and music performance in more than 50% of the participants.

Finally, the Gender PC included two PCs of acoustical descriptors, the first and the sixth, and explained 42.17% of the variance of the ranks of the Gender PC. The first PC of acoustical descriptors included the variable H2T_mea_, measuring the temporal separation between a heel and the consecutive toe strike; this could indicate that listeners expect female walkers to have smaller feet, i.e. shorter time interval between consecutive heel and toe strikes. The sixth PC included the variables H.mod_mea_ and T.mod_mea_, measuring the frequency of the most intense spectral component of heel and toe strike impact sounds; this could indicate that listeners expect female walkers to strike harder due to either smaller or harder shoe sole (e.g., high heels). As revealed by the inspection of the partial *R* ^2^ values, the most perceptually relevant variable for the rating scales reduced in the Gender PC was the sixth PC of acoustical descriptors. Notably, none of the PCs of acoustical descriptors significantly associated with the Gender PC included acoustical variables linking emotion expression in walking and music performance.

**Figure 4:**
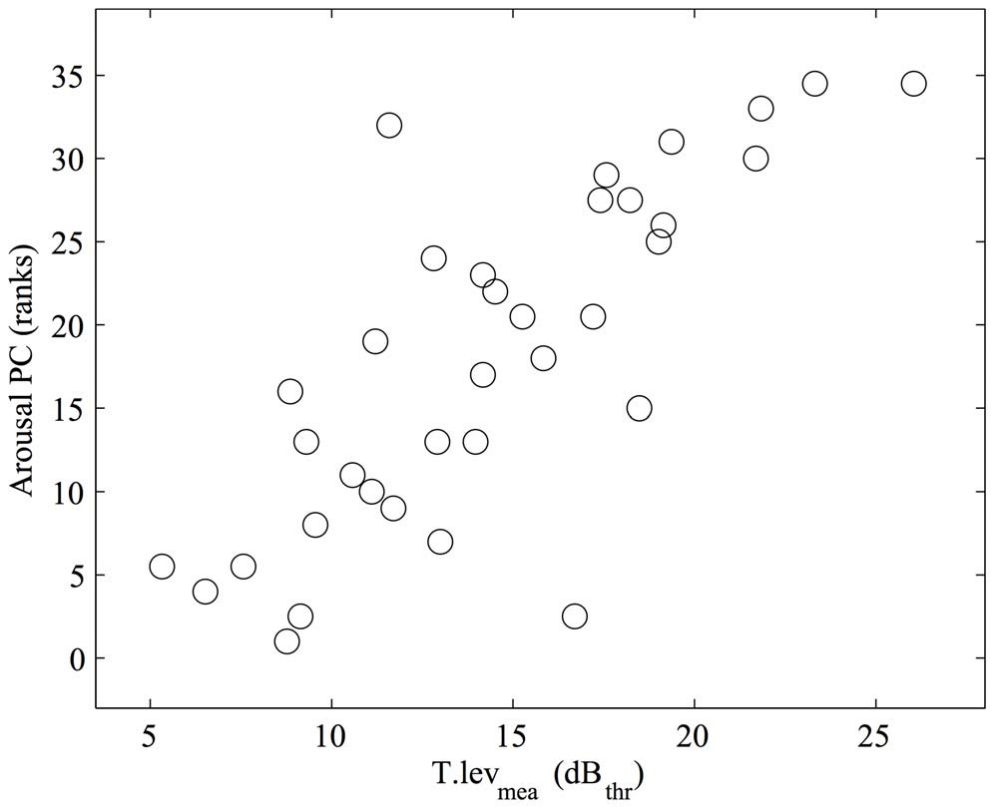
Acoustical correlates of the Arousal Principal Component (PC) of rating scales. Higher Arousal values result from higher ratings along the “Angry walker” scale, and from lower ratings along the “Sad walker” scale. T.lev_mea_ measures the average peak level of the toe strike impact sounds within the walking sound, measured in dB relative to the threshold level of the background noise.

**Figure 5:**
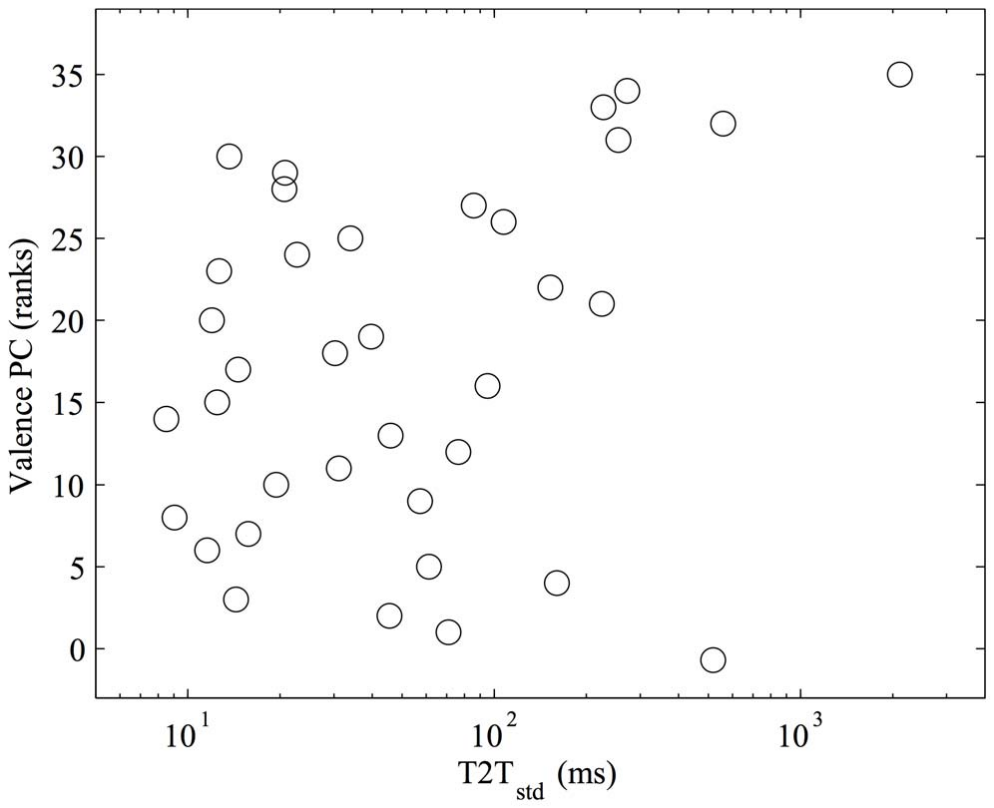
Acoustical correlates of the Valence Principal Component (PC) of rating scales. Higher Valence values result from higher ratings along the “Happy walker” scale, and from lower ratings along the “Fearful walker” and “Normal walker” scale. T2T_std_ measures the standard deviation of the temporal distance between the peak of temporally adjacent toe strike impact sounds within the walking sound. Higher T2T_std_ values emerge for walking sounds with a highly irregular pace.

**Figure 6:**
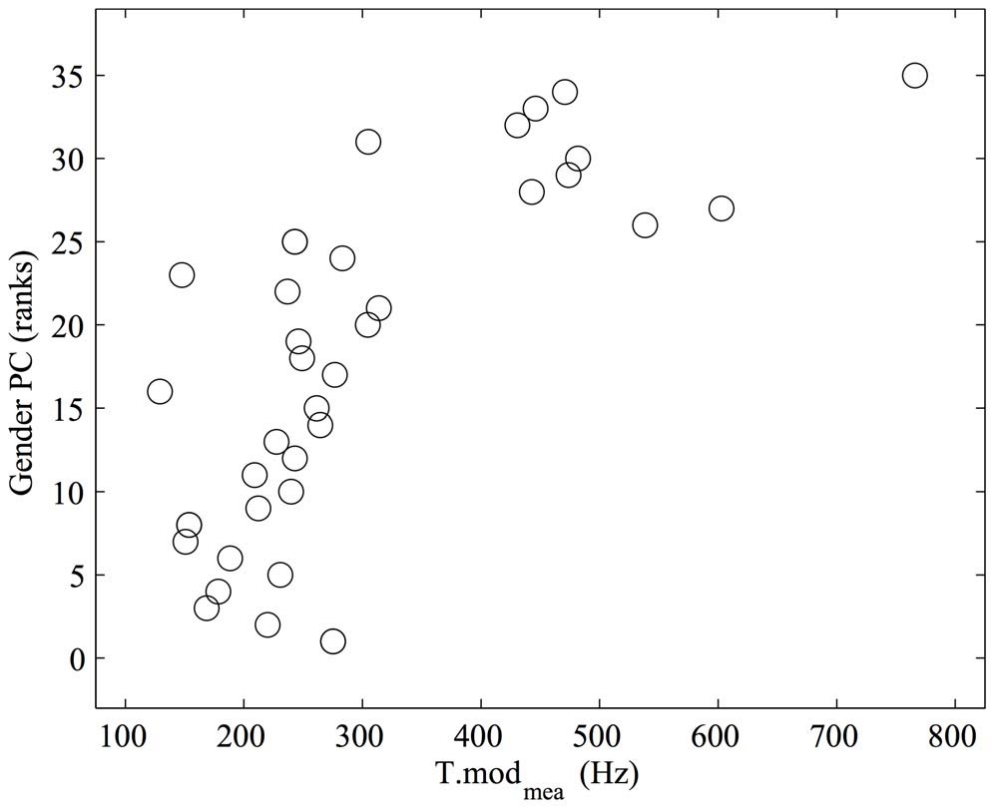
Acoustical correlates of the Gender Principal Component (PC) of rating scales. Higher Gender values result from higher ratings along the “Female walker”, “Light walker”, “Small sole” and “Hard sole” scales, and from lower ratings along the “Male walker”, “Heavy walker”, “Large sole” and “Soft sole” scales. T.mod_mea_ measures the frequency of the most intense spectral components of toe strike impact sounds, averaged within the walking sound.

Scatter plots relating unreduced acoustical variables to the PCs of rating scales are shown in Figs. 4–6. It emerges that arousal is strongly correlated with sound level (Fig. 4), valence with tempo variations (Fig. 5).

## General Discussion and Conclusions

Experiment 1 tested for similar feature use in emotionally-expressive walking and music performance, and revealed strong similarities between the two domains. Features representing sound intensity, tempo and tempo regularity emerged as been used similarly in the two domains to express emotion. In Experiment 2, listeners were able to recognize non-emotional and, with the exception of the fear and normal walking styles, emotional properties of the walkers. Taken together, these results support the motor origin hypothesis of the musical expression of emotions. However, we would like to add the motor hypothesis explains only part of the musical expression of emotions as it might exploit also sources of acoustical variability not available in non-vocal sounds, like frequency contours. Interestingly, the stronger point of contact between music and walking from both the performance and perceptual point of view appear to be temporal and energetic (sound intensity) rather than spectral acoustical information.

Experiment 2 also allowed to compare the recognition of emotional properties to emotion-independent properties like size, sole hardness, weight, and gender. Except for gender and fear recognition, the ability to recognize all other emotions appears to be equally developed. We also quantified the acoustical features relevant to the perception of emotions and of emotion-independent properties. Interestingly, the acoustics of the perception of emotion-independent properties of the walking event does not rely on variables linking walking and music performance. There could be therefore a separation on the perceptual use of acoustical parameters; some parameters (tempo and sound level) are used by listeners to identify information related to the expression of emotions, other parameters are instead used for the identification of emotion-independent properties of the walking sound source. Here for example, we replicated the results of Li, Logan and Pastore [24] that reported spectral properties to be of primary relevance to gender recognition.

Different than expected, the Valence PC model reveals that participants assigned higher happiness ratings to walking events with a higher pace variability. This finding does not correspond with previous findings where happiness was associated with lower tempo variability [6], and could be probably responsible for the extremely low performance in the recognition of this emotion. However, the regression model for the valence dimension is characterized by a particularly low ability to explain observed ratings. This fact could be explained in two ways. Firstly, the acoustical analyses may have captured not all of the signal features relevant to perception. Secondly, participants might have had cognitive limitations that did not allow them to reliably estimate three independent dimensions of judgment (e.g., arousal, valence and gender) within the one single experimental context. As such, listeners most likely focused on the most salient dimensions of variation within the experimental set: arousal and emotion-independent properties of the walking event. This latter hypothesis is more plausible than the former, and opens an interesting possibility: Emotional valence might be, within the domain of the acoustics of motor behavior, perceptually secondary.

Walkers in Experiment 1 were not musically trained, and emotion recognition in Experiment 2 was mostly independent of musical expertise. Thus, the similarities observed between emotion expression in walking and music performance may be caused by the fact that expression and recognition in music performance draws on previous knowledge of expressive body sounds and not the other way around. However, the findings presented in both experiments cannot provide a causal proof for motor-related musical emotion expression and recognition as only correlational evidence was presented here. The reported similarities between emotion expression in walking and music could of course be linked to any other non-observed variable not associated with motor activity. Further empirical evidence for a causal relationship between emotion expression and motor activity in music could be provided by showing motor-area co-activations during music listening (e.g. [41]), or including a control group without much motor experience in the expression of emotions in walking, such as paraplegics. Furthermore, additional research from a developmental or inter-cultural perspective may help to understand if the similarities observed across domains are innate or based on associative learning between walking and expressive auditory correlates.

Two experiments investigated acoustical feature use in expression and recognition of emotion in walking. Several features related to the timing and level of the walking sounds were identified as being consistently used in both experiments for expression and recognition and showed a high similarity to those features used for emotion expression and recognition in music performance. Even though the findings presented are subject to limitations, they are among the first to support the motor-origin hypothesis of musical emotion expression that states that musicians and listeners make use of general movement knowledge when expressing and recognizing emotions in music. That way the theory of vocal-origin of musical expression was extended [6] by showing that the reported acoustical patterns of emotion expression can also be found in other forms of human behavior.

## Supporting Information

S1 Table **Walker–by–walker spearman rank correlation coefficients between acoustical features used in the expression of emotions in the music and walking performance domains.** Correlation scores of -1, 0 and 1 indicate perfect dissimilarity, no similarity and perfect similarity, respectively.
(PDF)

S2 Table **Listener–by–listener performance in the recognition of the investigated properties of the walking events.** Spearman rank correlation coefficients of 1, 0 and -1 indicate perfect recognition, chance recognition and perfect mis–recognition, respectively.
(PDF)
