# Supplementary material for: The Production and Perception of Emotionally Expressive Walking Sounds: Similarities between Musical Performance and Everyday Motor Activity
Source: PLoS One. 2014 Dec 31;9(12):e115587. doi: 10.1371/journal.pone.0115587 (PMC4281241; doi:10.1371/journal.pone.0115587)
Supplement: S1 Table — Walker–by–walker spearman rank correlation coefficients between acoustical features used in the expression of emotions in the music and walking performance domains. Correlation scores of -1, 0 and 1 indicate perfect dissimilarity, no similarity and perfect similarity, respectively. (PDF) [file pone.0115587.s001.pdf]

Table S1

Walker–by–walker spearman rank correlation coefficients between acoustical features used in the expression of emotions in the music and walking performance domains. Correlation scores of -1, 0 and 1 indicate perfect dissimilarity, no similarity and perfect similarity, respectively.

Acoustical feature	Walker						
	1	2	3	4	5	6	7
$T2T_{mea}$	.73***	.75***	.25	.59**	.37	.75***	.25
$T2T_{std}$	.55**	−.01	.77***	.73***	−.16	−.12	.44*
$H.lev_{mea}$	.70***	.60**	.81***	.56**	.78***	.90***	.86***
$T.lev_{mea}$	.80***	.87***	.81***	.91***	.88***	.87***	.83***
$H.lev_{std}$	.37	.49*	−.31	−.39	.29	.63**	−.43
$T.lev_{std}$	.27	−.03	−.19	−.01	−.21	.57**	−.39
$H.SCG_{mea}$	−.47	−.31	−.80	−.19	−.55	−.80	−.45
$T.SCG_{mea}$	−.45	−.31	−.74	−.88	−.81	−.62	−.43
Art_{mea}	−.65	−.73	−.25	−.65	−.27	−.75	−.25
Art_{std}	.54*	.56**	−.43	−.19	−.04	−.04	−.13

*Note. H = Heel; T = Toe; lev = level; SCG = Spectral Center of Gravity; Art = articulation; mea = mean; std = standard deviation. * $p < .05$; ** $p < .01$; ***$p < .001$; p–values test the unidirectional hypothesis walking–music similarity >0 .*
