# Supplementary material for: The Production and Perception of Emotionally Expressive Walking Sounds: Similarities between Musical Performance and Everyday Motor Activity
Source: PLoS One. 2014 Dec 31;9(12):e115587. doi: 10.1371/journal.pone.0115587 (PMC4281241; doi:10.1371/journal.pone.0115587)
Supplement: S2 Table — Listener–by–listener performance in the recognition of the investigated properties of the walking events. Spearman rank correlation coefficients of 1, 0 and -1 indicate perfect recognition, chance recognition and perfect mis–recognition, respectively. (PDF) [file pone.0115587.s002.pdf]

Table S2

Listener–by–listener performance in the recognition of the investigated properties of the walking events. Spearman rank correlation coefficients of 1, 0 and -1 indicate perfect recognition, chance recognition and perfect mis–recognition, respectively.

Rating scale	Listener												
	1	2	3	4	5	6	7	8	9	10	11	12	13
Anger	.37*	.21	.37*	.21	.04	.28	.45**	.49**	.40**	.2	.23	.30*	.30*
Happiness	.35*	.38*	.41**	.59***	.39**	.18	.37*	.27	.37*	.41**	.15	−.01	.50**
Fear	.17	.23	−.03	.11	.16	−.06	.32*	.07	.42**	.22	.28	.24	.05
Normality	.15	.25	.2	.04	.21	.33*	.29*	−.02	.01	.22	.21	.01	.27
Sadness	.47**	.55***	.50**	.36*	.33*	.28	.59***	.20	.33*	.41**	.36*	.38*	.23
Gender	.30*	.75***	.65***	.72***	.54***	.76***	.59***	.74***	.43**	.72***	.70***	.45**	.48**
Weight	.17	.38*	.46**	.12	.05	.38*	.28	.41**	.39**	.51***	.56***	.53***	.29*
Size	.11	.55***	.82***	.46**	.50**	.56***	.49**	.64***	.27	.69***	.69***	.58***	.50**
Hardness	.47**	.76***	.89***	.24	.62***	.77***	.78***	.78***	.15	.55***	.79***	.68***	−.54

Note. * $p < .05$; ** $p < .01$; ***$p < .001$; p–values test the unidirectional hypothesis recognition performance > 0.
